# XBP1-FoxO1 interaction regulates ER stress-induced autophagy in auditory cells

**DOI:** 10.1038/s41598-017-02960-1

**Published:** 2017-06-30

**Authors:** Akihiro Kishino, Ken Hayashi, Chiaki Hidai, Takeshi Masuda, Yasuyuki Nomura, Takeshi Oshima

**Affiliations:** 1 Department of Otolaryngology, School of Medicine, Nihon University, Tokyo, 173-8610 Japan; 2 Department of Otolaryngology, Kamio Memorial Hospital, Tokyo, 101-0063 Japan; 3 Department of Physiology, School of Medicine, Nihon University, Tokyo, 173-8610 Japan

## Abstract

The purpose of this study was to clarify the relationship among X-box-binding protein 1 unspliced, spliced (XBP1u, s), Forkhead box O1 (FoxO1) and autophagy in the auditory cells under endoplasmic reticulum (ER) stress. In addition, the relationship between ER stress that causes unfolded protein response (UPR) and autophagy was also investigated. The present study reported ER stress induction by tunicamycin treatment that resulted in IRE1α-mediated XBP1 mRNA splicing and autophagy. XBP1 mRNA splicing and FoxO1 were found to be involved in ER stress-induced autophagy. This inference was based on the observation that the expression of LC3-II was suppressed by knockdown of IRE1α, XBP1 or FoxO1. In addition, XBP1u was found to interact with XBP1s in auditory cells under ER stress, functioning as a negative feedback regulator that was based on two important findings. Firstly, there was a significant inverse correlation between XBP1u and XBP1s expressions, and secondly, the expression of XBP1 protein showed different dynamics compared to the XBP1 mRNA level. Furthermore, our results regarding the relationship between XBP1 and FoxO1 by small interfering RNA (siRNA) paradoxically showed negative regulation of FoxO1 expression by XBP1. Our findings revealed that the XBP1-FoxO1 interaction regulated the ER stress-induced autophagy in auditory cells.

## Introduction

Cells are continuously exposed to not only external stress such as starvation, ischemia and oxidative stress, but also intracellular stress like endoplasmic reticulum (ER) stress. ER is an essential subcellular organelle responsible for protein folding and secretion^1, 2^. ER stress is caused by the accumulation of unfolded or misfolded proteins in ER and induces an adaptive mechanism known as the unfolded protein response (UPR) or ER stress response^3, 4^. In order to restore ER homeostasis, UPR activates the transcription of several genes involved in the reduction of protein synthesis, ER-associated protein degradation (ERAD) and ER chaperons^5^. However, UPR failure results in cell death.

In mammalian cells, three major ER stress sensors have been identified: Inositol-requiring protein1α (IRE1α), PKR-like ER kinase (PERK) and activating transcription factor 6 (ATF6)^6–8^. Under ER stress, these proteins initiate the UPR signaling cascades to alleviate the burden of unfolded proteins. Of these three major ER stress sensors, IRE1α signaling pathway is the most evolutionarily conserved from yeast to mammals. IRE1α is a transmembrane RNase involved in X-box-binding protein 1 (XBP1) mRNA splicing^9, 10^. XBP1 is a major regulator of UPR, mediating adaptation to ER stress. XBP1 has two isoforms, i.e. XBP1 spliced (s) and XBP1 unspliced (u). XBP1s is a key transcriptional factor that regulates the transcription of genes involved in UPR. XBP1u is an inactivate form with no transcriptional activity^11^. IRE1α is activated by dimerization and autophosphorylation under ER stress condition^12^. XBP1u mRNA is produced constitutively and yields an unstable protein XBP1u, which undergoes rapid proteasomal degradation by the proteasome^13^. ER stress allows phosphorylated IRE1α (p-IRE1α) to remove a 26 nucleotides intron from XBP1u mRNA by cytoplasmic splicing on the ER membrane, inducing a shift in the open reading frame^14^. To promote transcription, XBP1s mRNA is translated into protein XBP1s, which moves into the nucleus and binds to the UPR element in the gene transcription space required for the UPR and ERAD^9, 15^.

Recent findings indicated that ER stress was involved in the pathogenesis of neurodegenerative diseases, psychiatric diseases and aging^16–18^, and also caused sensorineural hearing loss^19–21^ or age-related hearing loss^22^. Additionally, it has been reported that XBP1 impairment contributes to not only neurodegenerative disorders including Parkinson’s and Alzheimer’s disease but also metabolic disorders, inflammatory disease, and cancers^23–43^. Oishi *et al*., using the mouse model, suggested that XBP1 deficiency contributed to aminoglycoside-induced sensorineural hearing loss^6^.

In addition, it has been found that IRE1α signaling could mediate the connection between the UPR and autophagy through XBP1 mRNA splicing to degrade accumulated unfolded or misfolded proteins and thus alleviate ER stress^44^. Autophagy is an intracellular degradation process by which cytoplasmic constitutions are delivered to the lysosome for the maintenance of homeostasis and bioenergetics in the mammalian cells, and also the cell death or premature senescence of auditory cells^45, 46^. It has been reported that autophagy has two pathways of prosurvival functions and cell death under different physiological and pathological conditions. Autophagy is rarely and persistently activated in response to stress to avoid autophagic cell death, but the excessive induction of autophagy results in cell death^47^. The dysfunction of autophagy induces various disorders including neurodegeneration or aging^48^.

Forkhead box O1 (FoxO1) is a transcriptional factor, which is involved in several important biological processes, such as cell-cycle arrest, apoptosis and aging^49, 50^. Recent reports described the involvement of FoxO1 in the induction of autophagy through cytosolic or transcriptional activity in neurocyte and human cancer cell lines^51–53^. Besides, a number of studies demonstrated the potential interaction of FoxO1 with XBP1. Zhao *et al*. reported that XBP1u suppresses autophagy by degradation of FoxO1 through 20S proteasome in the cancer cells^54^. While Zhou *et al*. reported that XBP1s negatively regulates FoxO1 by proteasome-mediated degradation in pancreatic β cells^55^. Although previous reports demonstrated FoxO1 has key functions in the regulation of autophagy^56^, the mechanisms linking XBP1 with the modulation of FoxO1 are not fully understood in case of auditory cells.

The relationship between ER stress signaling pathway and autophagy remains unclear in the case of auditory cells. ER stress has been reported to induce cellular dysfunction and apoptotic cell death^19^. Tunicamycin, an inhibitor of N-acetylglucosamine transferase, is one of widely used ER stress inducer^57^. Tunicamycin inhibits N-linked glycosylation of immature proteins^58^. Blockage of N-linked glycosylation results in the accumulation of misfolded proteins in ER and ultimately prolonged stress induces cell death^59^. Previous report demonstrated that the treatment of tunicamycin induced the expression of C/EBP homologous protein (CHOP), a specific ER stress-associated pro-apoptotic factor, in hair cells and spinal ganglion cells (SGCs) in cochlea, and caused degeneration of hair cells and SGCs^19, 20^. The cell death induced by tunicamycin in auditory cells showed the characteristics of apoptotic cells^21^. However, it is difficult to explain only from the perspective of cellular level apoptosis, because the hearing level varies widely and is not permanent. Therefore, it is necessary to analyze the inner ear protective efficacy from both the perspectives of UPR and autophagy. The correlation between UPR and autophagy remains unclear in auditory cells under ER stress. We believe that the investigation of the ER stress in auditory cells can make a major contribution to the understanding of the mechanisms of inner ear diseases including sensorineural hearing loss, and lead to the development of novel therapeutics. On the basis of these reasons, we analyzed the influence of ER stress on auditory cells by focusing on the function of XBP1, FoxO1 and autophagy. The purpose of this study was to consider the function of XBP1 and FoxO1 in auditory cells under ER stress condition, and the correlation of UPR and autophagy.

## Results

### Tunicamycin treatment induces both late apoptotic and necrotic cell death in auditory cells

**Figure 1:**
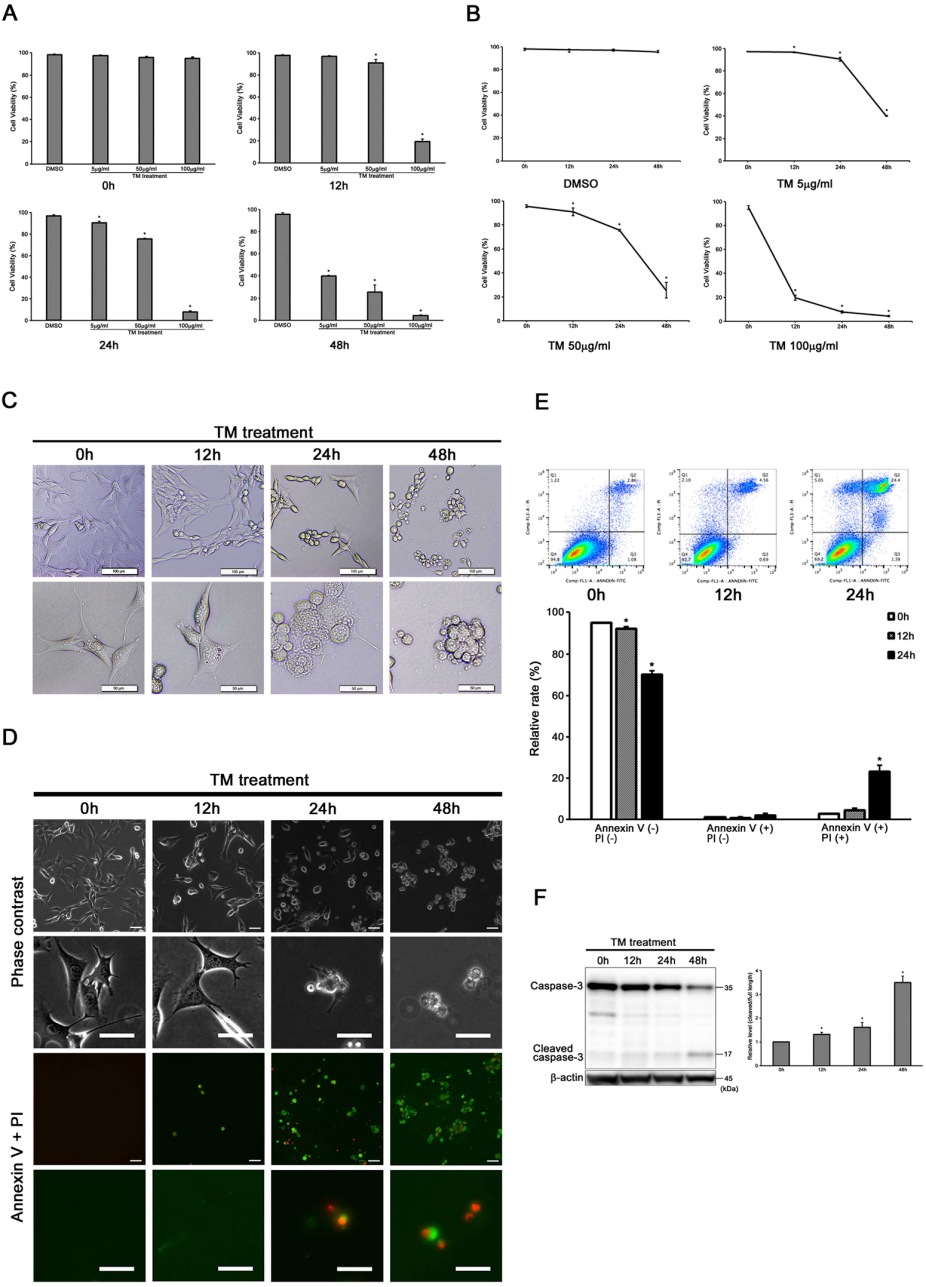
Tunicamycin treatment induces both apoptotic and necrotic cell death in auditory cells. (**A**) Cell viability was decreased in a dose-dependent manner in tunicamycin-treated HEI-OC1 cells. Cells were treated with different concentrations of tunicamycin (5, 50 and 100 µg/ml) for 12, 24 and 48 h. (**B**) Cell viability was decreased in time-dependent manner in tunicamycin-treated HEI-OC1 cells. (**C**) Cells were treated with 50 µg/ml tunicamycin for designed periods of time (12, 24 and 48 h). Cells morphology was observed under light microscopy. The structure of nucleus was normal in the cells with sharp cell process (50 µg/ml for 0 and 12 h). Cell membrane was swollen, and nucleus was enlarged in tunicamycin-treated HEI-OC1 cells (50 µg/ml for 24 h). The cell membrane breaks down with extraction cellular contents (50 µg/ml for 48 h). (**D**) Representative fluorescence microscopy images of cells treated with tunicamycin (50 µg/ml) for designed periods of time (12, 24 and 48 h) stained with Annexin V and PI. Scale bars, 50 µm. (**E**) Flow cytometry showed the increased populations at late apoptosis and at necrosis (Annexin V+, PI+). The data in (**A**), (**B**) and (**E**) are shown as means ± S.D. of three or more independent studies (*p < 0.05 versus control group). (**F**) Representative Western blots showing the expressions of full length and cleaved caspase-3. β-actin was included as a loading control. The expressions of full length and cleaved caspase-3 was detected, and the means ± S.D. (fold of changes over the control group) of three or more independent studies were presented (*p < 0.05 versus control group). Full-length blots are presented in Supplementary Figure 1a and b.

We analyzed the cell viability after treatment with different concentrations of tunicamycin (0, 5, 50 and 100 µg/ml) for 0, 12, 24 and 48 h, respectively, to examine the effect of ER stress on the HEI-OC1 cells. As shown in Fig. 1A and B, the HEI-OC1 cells treated with tunicamycin exhibited dose- and time-dependent cell death. Exposure to tunicamycin decreased the cell viability up to 25.6% at 50 µg/ml for 48 h.

Next, we observed the cell morphology under light microscopy. Both the cell membrane and the nucleus were swollen at 24 h after treatment, and eventually, the cell membrane was ruptured, and cytoplasm was discharged at 48 h. These morphological changes indicated necrosis-like morphology (Fig. 1C).

Cells were subjected to Annexin V-FITC and PI staining, followed by cytometry analysis in order to confirm whether tunicamycin exposure induces apoptosis or necrosis in the HEI-OC1 cells (Fig. 1D). As shown in Fig. 1E, tunicamycin exposure induced both late apoptotic and necrotic cell death.

Then, we evaluated the expression of full length and cleaved caspase-3 by Western blot analysis in order to confirm whether tunicamycin exposure activated the apoptotic signaling pathway in the HEI-OC1 cells. As shown in Fig. 1F, the expression of cleaved caspase-3 increased and that of full length of caspase-3 decreased gradually.

These results suggested that tunicamycin-induced ER stress induces both late apoptotic and necrotic cell death.

### ER stress induces XBP1 mRNA splicing in auditory cells

**Figure 2:**
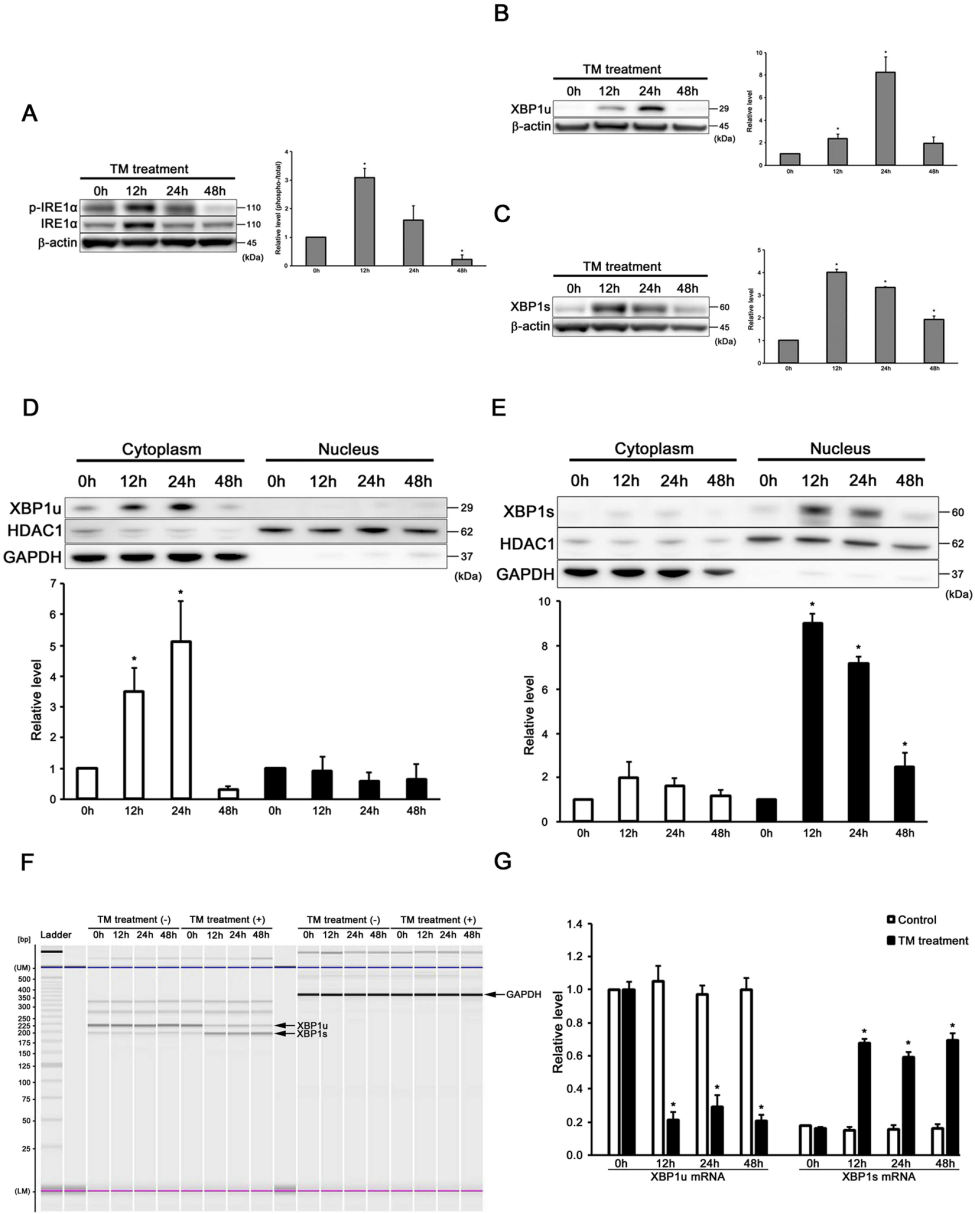
ER stress induces XBP1 mRNA splicing in auditory cells. (**A**–**C**) Representative Western blots showing the expressions of (**A**) p-IRE1α, IRE1α, (**B**) XBP1u and (**C**) XBP1s. β-actin was included as a loading control. The expressions of p-IRE1α, IRE1α, XBP1u and XBP1s were detected, and the means ± S.D. (fold of changes over the control group) of three or more independent studies were presented (*p < 0.05 versus control group). Full-length blots are presented in Supplementary Figure 2a–f. (**D**,**E**) Western blots showing cytoplasmic and intranuclear XBP1u and XBP1s expressions at the designated time after tunicamycin treatment (50 µg/ml for 48 h). GAPDH was included as a loading cytoplasm control, and HDAC1 was used as a nuclear control. The expression of XBP1u was detected only in the cytoplasm. The expression of XBP1s was detected only in the nucleus. The means ± S.D. (fold of changes over the control group) of three or more independent studies were presented (*p < 0.05 versus control group). Full-length blots are presented in Supplementary Figure 2g–l. (**F**,**G**) RT-PCR analysis showing the expression of XBP1u mRNA and XBP1s mRNA. Cells were treated with or without 50 µg/ml tunicamycin for designed periods (12, 24 and 48 h), and the total RNA was extracted and subjected to RT-PCR analysis. GAPDH was included as a loading control. “UM” and “LM” each indicate the upper and lower markers, respectively. The expressions of XBP1u mRNA and XBP1s mRNA were detected, and the means ± S.D. (fold of changes over the control group) of three independent studies were presented (*p < 0.05 versus control group).

We evaluated the expressions of p-IRE1α, IRE1α, XBP1u and XBP1s by Western blot analysis in order to confirm that ER stress induces XBP1 mRNA splicing in the auditory cells. The expression levels of p-IRE1α and IRE1α peaked at 12 h and decreased at 24 h after treatment (Fig. 2A). The expression level of XBP1u peaked at 24 h and decreased at 48 h after the treatment (Fig. 2B). The expression level of XBP1s peaked at 12 h and gradually decreased from 24 h after the treatment (Fig. 2C). These results indicate that XBP1 mRNA splicing process is likely to be associated with activation of IRE1α in auditory cells.

Then, we examined the expression of intranuclear and cytoplasmic XBP1u, s protein level by the Western blotting technique in order to evaluate whether XBP1u, s translates into the nuclei in HEI-OC1 cells under ER stress. As shown in Fig. 2D, the expression level of cytoplasmic XBP1u peaked at 24 h after treatment with tunicamycin, but that of intranuclear XBP1u could not be detected. On the other hand, the expression level of intranuclear XBP1s peaked at 12 h after the treatment; however, that of cytoplasmic XBP1s could not be detected (Fig. 2E). These results suggest that XBP1u was mainly localized in the cytoplasm and XBP1s in the nucleus of the auditory cells.

Next, we examined RT-PCR analysis to detect XBP1 mRNA splicing in the auditory cells under ER stress condition. As shown in Fig. 2F and G, the HEI-OC1 cells without tunicamycin treatment showed a low level of XBP1s mRNA and a high level of XBP1u mRNA. The expression level of XBP1s mRNA markedly increased in the case of tunicamycin-treated cells and remained high from 12 h after treatment. On the other hand, the expression level of XBP1u mRNA decreased and remained low from 12 h after the tunicamycin treatment. These observations suggest that ER stress induces XBP1 mRNA splicing in the auditory cells.

### ER stress induces autophagy in auditory cells

**Figure 3:**
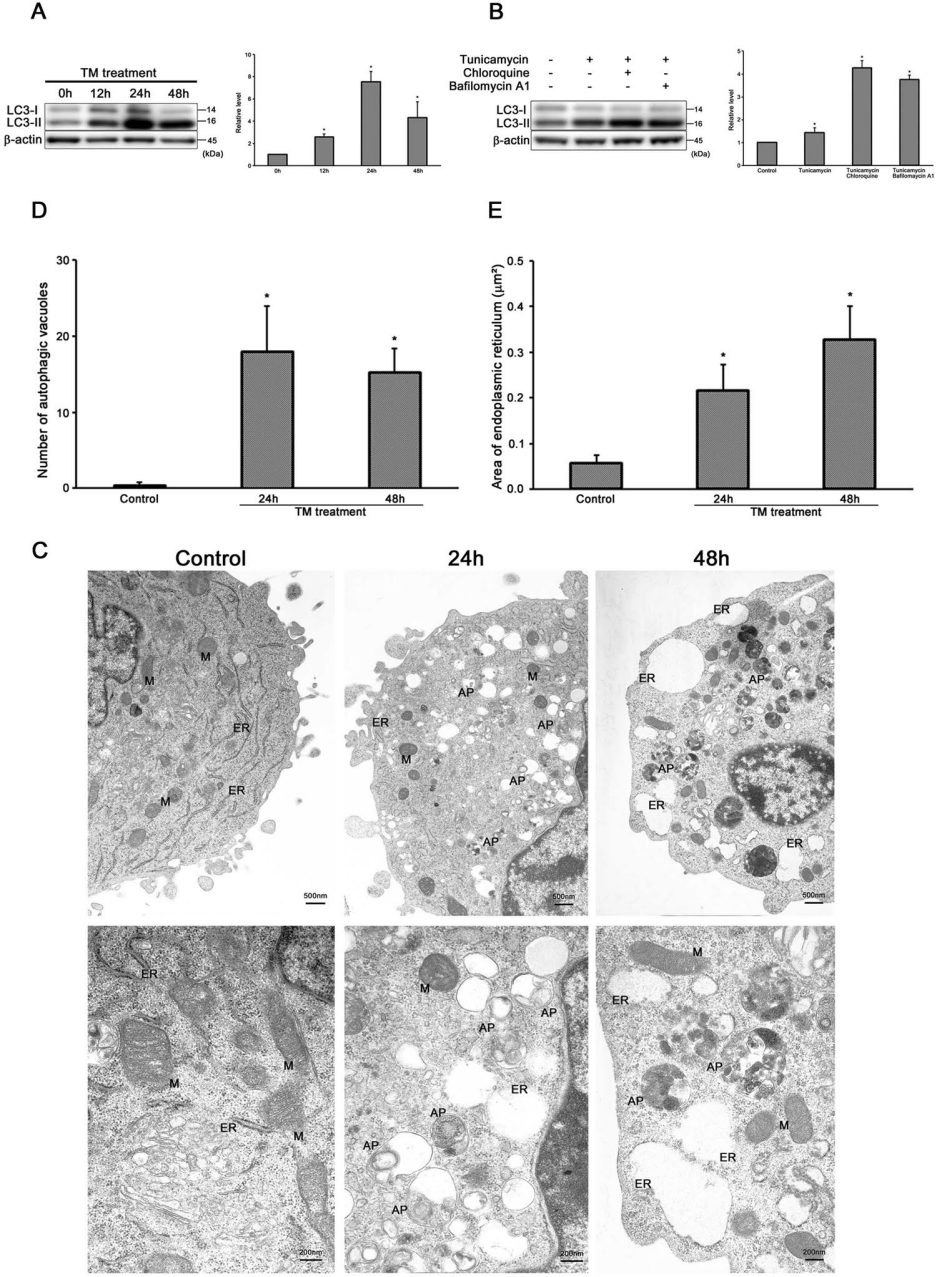
ER stress induces autophagy in auditory cells. (**A**) Representative Western blots showing the expressions of LC3-II. β-actin was included as a loading control. The expression of LC3-II was detected, and the means ± S.D. (fold of changes over the control group) of three or more independent studies were presented (*p < 0.05 versus control group). Full-length blots are presented in Supplementary Figure 3a and b. (**B**) Cells were treated with 50 µg/ml tunicamycin in the presence of chloroquine (50 µM) or bafilomycin A1 (100 nM) for 24 h, and subjected to Western blots analysis. The expressions of LC3-II in tunicamycin-treated cells was markedly increased by lysosomal inhibitors. β-actin was included as a loading control. The expression of LC3-II was detected, and the means ± S.D. (fold of changes over the control group) of three or more independent studies were presented (*p < 0.05 versus control group). Full-length blots are presented in Supplementary Figure 3c and d. (**C**) Representative transmission electron microscopy shows morphological changes in HEI-OC1 cells treated with tunicamycin (50 µg/ml for 24 h and 48 h). The structure of the nucleus and organelles are normal in control cells (left panel, ×10,000). Treated cells with tunicamycin (50 µg/ml for 24 h) leads to the formation of autophagosome and mild ER expansion (middle panel, ×10,000). Treated cells with tunicamycin (50 µg/ml for 48 h) exhibits some autophagosomes and hyper ER expansion (right panel, ×10,000). M, mitochondria; ER, endoplasmic reticulum; AP, autophagosome. (**D**) The number of autophagic vacuoles was counted per one cell, which was presented as the mean ± S.D. from three or more independent experiments (*p < 0.05 versus control group). (**E**) The area of ER was measured per one ER, which was presented as the mean ± S.D. from three or more independent experiments (*p < 0.05 versus control group).

We evaluated the expressions of microtubule-associated protein 1 light chain 3-II (LC3-II) by Western blot analysis to investigate the relationship between ER stress and autophagy in auditory cells. The expression level of LC3-II peaked at 24 h and decreased at 48 h after the tunicamycin treatment (Fig. 3A). Importantly, tunicamycin-treated cells in the presence of chloroquine or bafilomycin A1, inhibitors of fusion between lysosome and autophagosome, showed further increase in LC3-II expression level (Fig. 3B). These results mean autophagy can be induced in tunicamycin-treated auditory cells.

Next, we examined the ultrastructural changes in auditory cells under ER stress condition by transmission electron microscopy (TEM). TEM revealed that cells treated with tunicamycin for 24 h displayed the formation of autophagosome, which was characteristic of autophagic response and the mild expansion of ER. At this point, autophagosomes contained not only organelle but also the contents like lipofuscin and aggregates. At 48 h after the treatment with tunicamycin, the cell membrane was conspicuously-enlarged with a shrinking nucleus. The expansion rate of ER was significantly increased more than 24 h in tunicamycin-treated cells (Fig. 3C). The number of autophagic vacuoles peaked at 24 h and then decreased at 48 h in tunicamycin-treated cells (Fig. 3D). There was a significant difference observed between the control and treated cells. The size of ER was time-dependently increased in tunicamycin-treated cells (Fig. 3E). These results show that ER stress by tunicamycin treatment induces autophagy in auditory cells.

### XBP1 participates in the induction of autophagy in auditory cells

**Figure 4:**
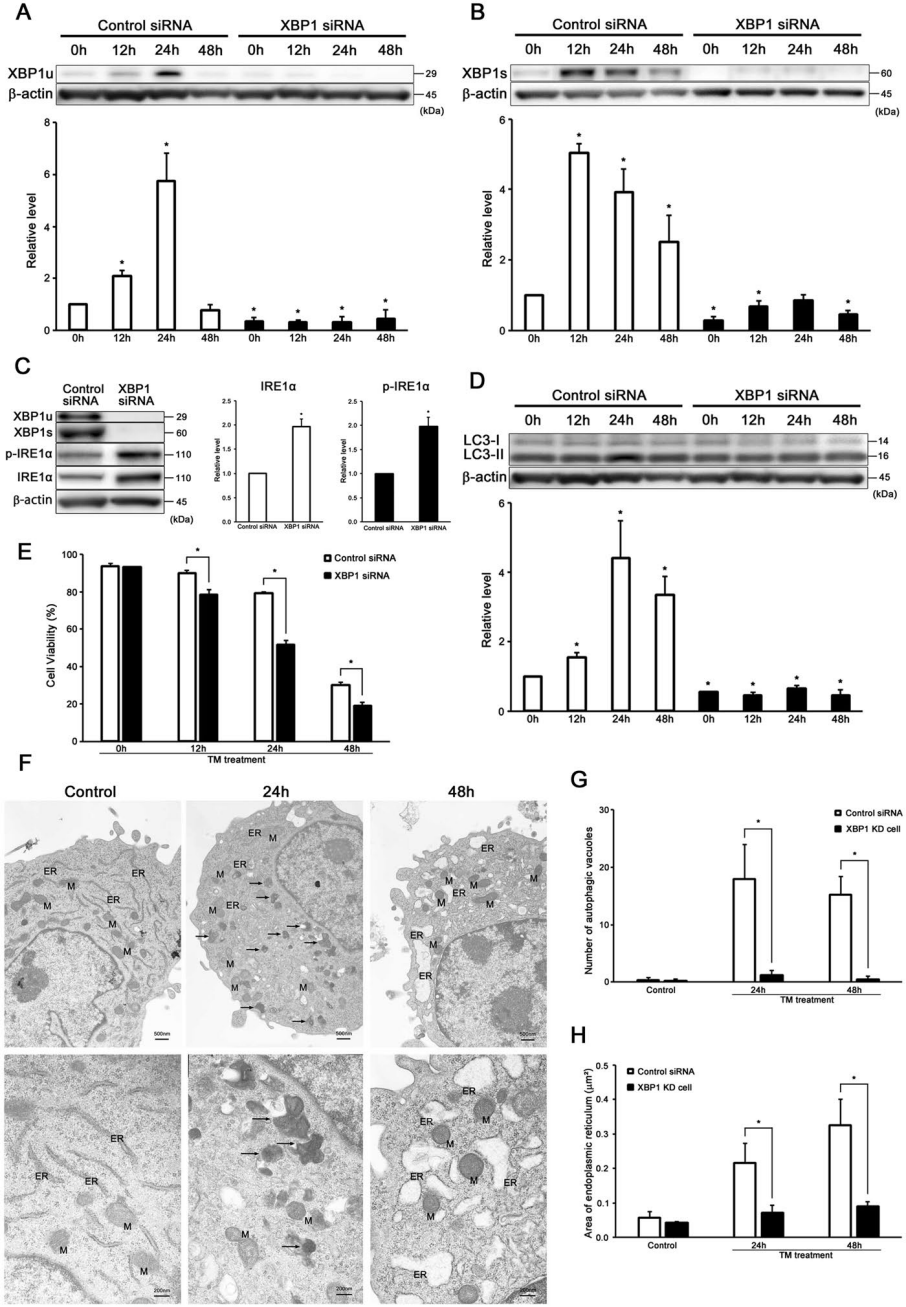
XBP1 participates in the induction of autophagy in auditory cells. (**A**–**D**) Knockdown of XBP1 suppressed the induction of LC3-II. β-actin was included as a loading control. The expressions of XBP1u, XBP1s, p-IRE1α, IRE1α and LC3-II were detected, and the means ± S.D. (fold of changes over the control group) of three or more independent studies were presented (*p < 0.05 versus control group). Full-length blots are presented in Supplementary Figure 4a–j. (**E**) After transmission with XBP1 and control siRNA for 48 h, the cells were treated with tunicamycin (50 µg/ml for 48 h), and cell viability was measured. The data are shown as means ± S.D. of three or more independent studies (*p < 0.05 versus control group). (**F**) Representative transmission electron microscopy shows morphological changes of XBP1 KD cells treated with tunicamycin (50 µg/ml for 24 h and 48 h). The structure of the nucleus and organelles are normal in the control cells (left panel, ×30,000). Lipofuscin and aggregates exist in XBP1 KD cells treated with tunicamycin (50 µg/ml for 24 h), although the autophagosome was not confirmed (middle panel, ×30,000). The rate of ER expansion was lower than the tunicamycin-treated cells in tunicamycin-treated XBP1 KD cells (50 µg/ml for 48 h) (right panel, ×30,000). M, mitochondria; ER, endoplasmic reticulum; AP, autophagosome. The arrows point to lipofuscin. (**G**) The number of autophagic vacuoles was counted per one cell, which was presented as the mean ± S.D. from three independent experiments (*p < 0.05 versus control group). (**H**) The area of ER was measured per one ER, which was presented as the mean ± S.D. from three or more independent experiments (*p < 0.05 versus control group).

XBP1 was knocked down (KD) by siRNA so as to examine the relationship between ER stress response and the induction of autophagy in auditory cells. The expressions of IRE1α and p-IRE1α were significantly increased in tunicamycin-treated XBP1 KD cells (Fig. 4A–C). The induction of LC3-II was significantly blocked in tunicamycin-treated XBP1 KD cells (Fig. 4D). Figure 4E shows that the cell viability was significantly decreased in tunicamycin-treated XBP1 KD cells (50 µg/ml for 48 h).

TEM revealed that the lipofuscin and aggregates existed in tunicamycin-treated XBP1 KD cells (50 µg/ml for 24 h), although the presence of autophagosome was not confirmed. As shown in Fig. 4F, ER expansion was increased time-dependently, but it was not as wider as that of tunicamycin-treated cells (50 µg/ml for 48 h). Besides, few materials like lipid were observed inside the ER. The number of autophagic vacuoles in tunicamycin- treated XBP KD cells was significantly less than tunicamycin-treated si-control cells (Fig. 4G). There was a significant difference in the size of ER between XBP1 KD cells and tunicamycin-treated si-control cells (Fig. 4H). These results showed that XBP1 participates in the induction of autophagy in auditory cells under ER stress condition.

### IRE1α-mediated XBP1 mRNA splicing is necessary for ER stress-induced autophagy in auditory cells

**Figure 5:**
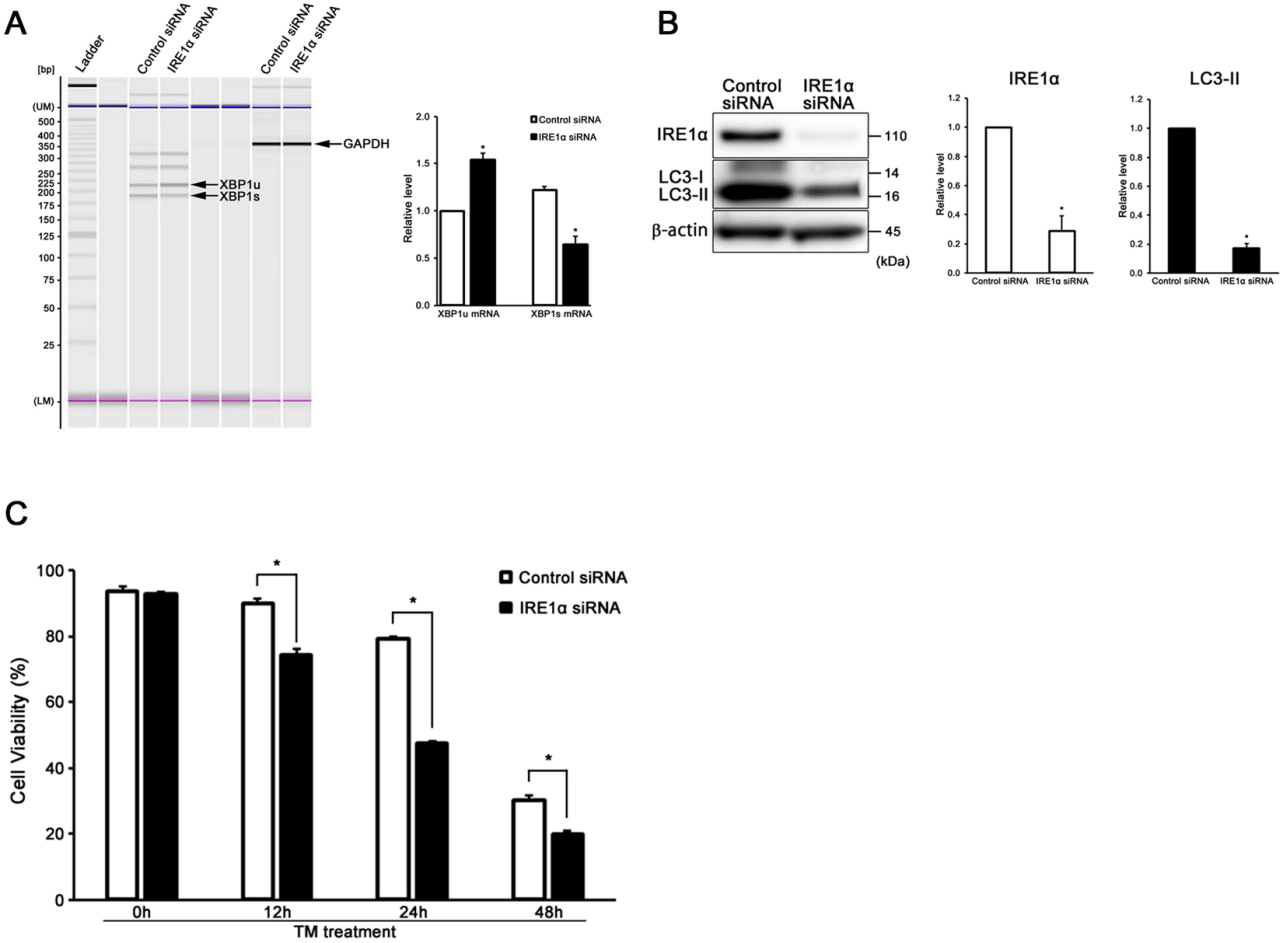
IRE1α-mediated XBP1 mRNA splicing is necessary for ER stress-induced autophagy in auditory cells. (**A**) RT-PCR analysis showing the expression of XBP1u mRNA and XBP1s mRNA in si-control cells and IRE1α KD cells. Cells were treated with 50 µg/ml tunicamycin for 24 h, and the total RNA was extracted and subjected to RT-PCR analysis. GAPDH was included as a loading control. “UM” and “LM” each indicate the upper and lower markers, respectively. The expressions of XBP1u mRNA and XBP1s mRNA were detected, and the mean ± S.D. (fold of changes over the control group) of three independent studies were presented (*p < 0.05 versus control group). (**B**) Knockdown of IRE1α suppressed the induction of LC3-II. β-actin was included as a loading control. The expressions of IRE1α and LC3-II were detected, and the mean ± S.D. (fold of changes over the control group) of three or more independent studies were presented (*p < 0.05 versus control group). Full-length blots are presented in Supplementary Figure 5a–c. (**C**) After transmission with IRE1α and control siRNA for 48 h, the cells were treated with tunicamycin (50 µg/ml for 48 h), and cell viability was measured. The data are shown as mean ± S.D. of three or more independent studies (*p < 0.05 versus control group).

We knocked down IRE1α by siRNA and then examined the expression of XBP1 at the mRNA level using RT-PCR to confirm whether IRE1α directly affected XBP1 mRNA splicing in auditory cells under ER stress condition. The expression level of XBP1s mRNA was significantly decreased, but that of XBP1u mRNA was significantly increased in IRE1α KD cells in comparison with the si-control cells at 24 h after tunicamycin treatment (Fig. 5A). These observations suggest that IRE1α has a direct influence on XBP1 mRNA splicing in auditory cells.

Next, we evaluated the expression of LC3-II in tunicamycin-treated IRE1α KD cells by the Western blot technique to investigate whether XBP1 mRNA splicing induced autophagy in auditory cells under ER stress. The induction of LC3-II was significantly blocked in tunicamycin-treated IRE1α KD cells (Fig. 5B). Figure 5C shows that the cell viability was significantly decreased in tunicamycin-treated IRE1α KD cells (50 µg/ml for 48 h). These results show that XBP1 mRNA splicing is necessary for ER stress-induced autophagy in auditory cells, and autophagy serves as a cell survival mechanism to protect against ER stress-induced cell death.

### FoxO1 is involved in autophagy in auditory cells under ER stress condition

**Figure 6:**
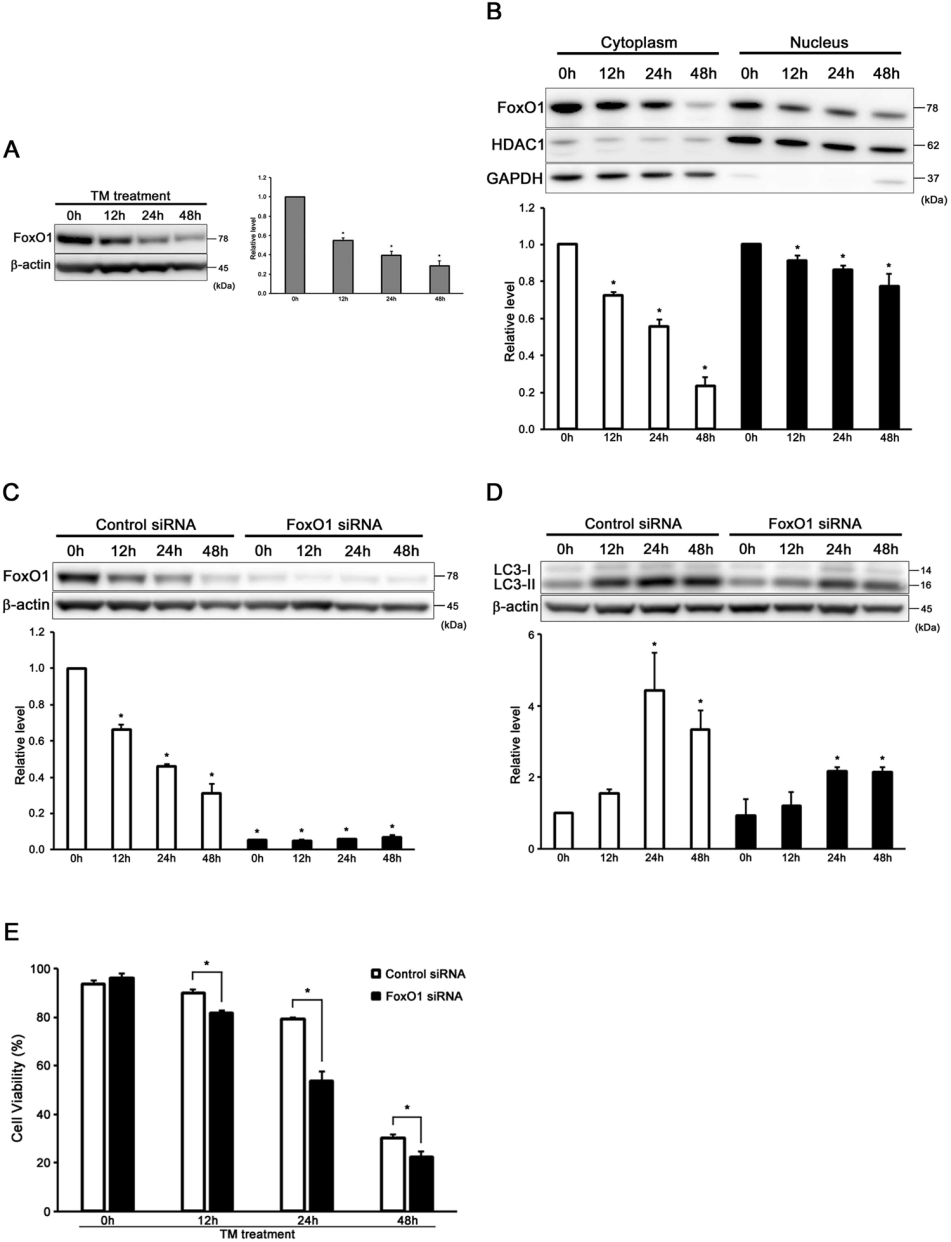
FoxO1 is involved in autophagy in auditory cells under ER stress condition. (**A**) Representative Western blots showing the expression of FoxO1. β-actin was included as a loading control. The expression of FoxO1 was detected, and the means ± S.D. (fold of changes over the control group) of three or more independent studies were presented (*p < 0.05 versus control group). Full-length blots are presented in Supplementary Figure 6a and b. (**B**) Western blots showing cytoplasmic and intranuclear FoxO1 expression at the designated time after tunicamycin treatment (50 µg/ml for 48 h). GAPDH was included as a loading cytoplasm control, and HDAC1 was used as a nuclear control. The expression of FoxO1 was detected both in cytoplasm and nucleus. The means ± S.D. (fold of changes over the control group) of three or more independent studies were presented (*p < 0.05 versus control group). Full-length blots are presented in Supplementary Figure 6c–e. (**C**,**D**) Knockdown of FoxO1 suppressed the induction of LC3-II. β-actin was included as a loading control. The expressions of FoxO1 and LC3-II were detected, and the mean ± S.D. (fold of changes over the control group) of three or more independent studies were presented (*p < 0.05 versus control group). Full-length blots are presented in Supplementary Figure 6f–h. (**E**) After transmission with FoxO1 and control siRNA for 48 h, the cells were treated with tunicamycin (50 µg/ml for 48 h), and cell viability was measured. The data are shown as mean ± S.D. of three or more independent studies (*p < 0.05 versus control group).

We evaluated the expression of FoxO1 in control cells and LC3-II in tunicamycin-treated FoxO1 KD cells by Western blot analysis to understand whether FoxO1 functions as a mediator of the induction of autophagy in auditory cells under ER stress. The expression of FoxO1 was decreased time-dependently in the control cells (Fig. 6A). Both the expressions of intranuclear and cytoplasmic FoxO1 were decreased time-dependently (Fig. 6B). The induction rate of LC3-II was significantly suppressed in FoxO1 KD cells in comparison with si-control cells (Fig. 6C and D). Figure 6E shows that the cell viability was significantly decreased in tunicamycin-treated FoxO1 KD cells (50 µg/ml for 48 h). These results suggest that FoxO1 is involved in ER stress-induced autophagy in auditory cells.

### XBP1 regulates the FoxO1 expression in auditory cells under ER stress condition

**Figure 7:**
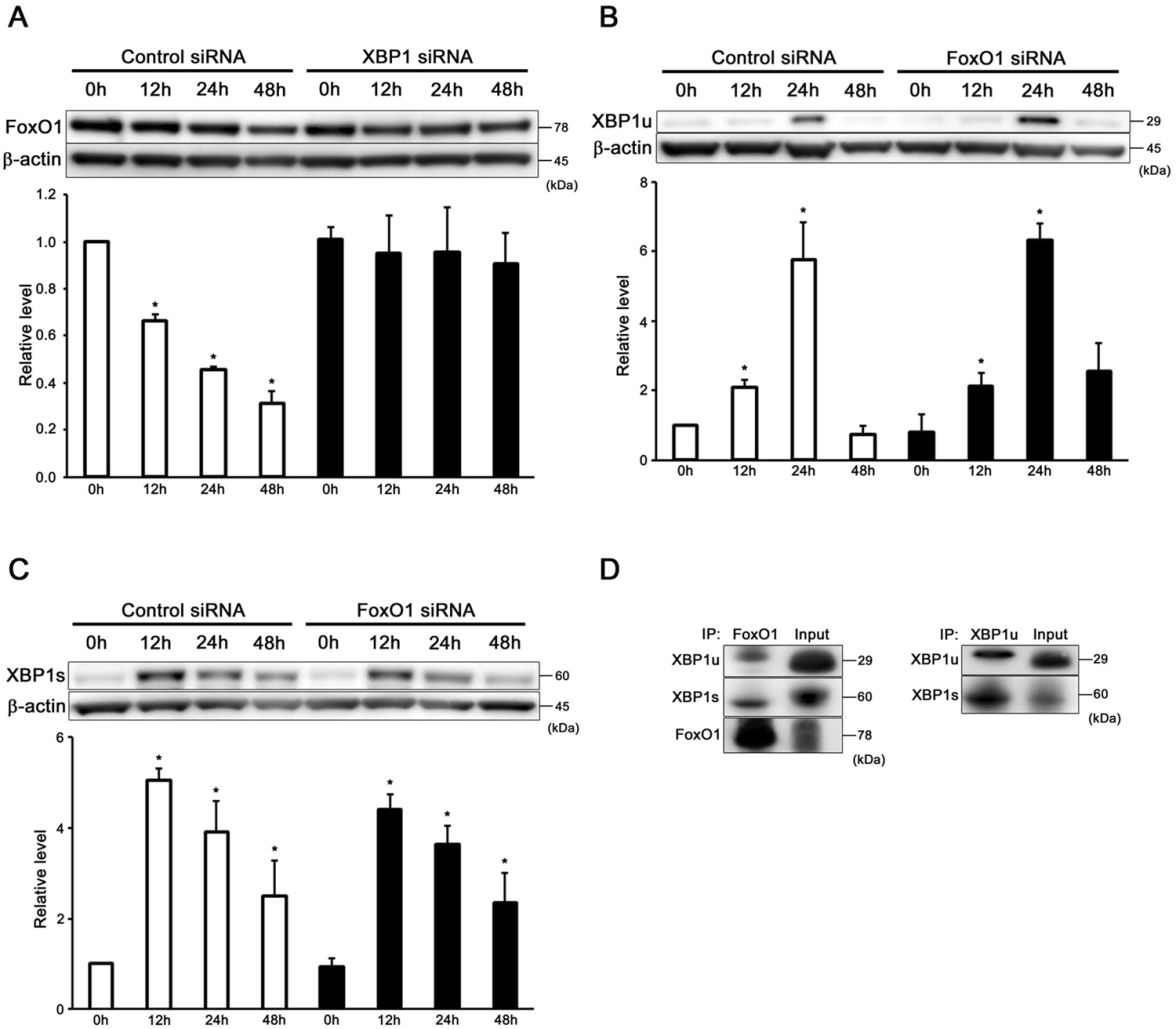
XBP1 regulates the FoxO1 expression in auditory cells under ER stress condition. (**A**) XBP1 KD cells showed no significant change in the FoxO1 expression. β-actin was included as a loading control. The expressions of FoxO1 was detected, and the mean ± S.D. (fold of changes over the control group) of three or more independent studies were presented (*p < 0.05 versus control group). Full-length blots are presented in Supplementary Figure 7a and b. (**B**,**C**) FoxO1 KD cells showed no significant difference of the XBP1u and XBP1s expressions in comparison with si-control cells. β-actin was included as a loading control. The expressions of XBP1u and XBP1s were detected, and the means ± S.D. (fold of changes over the control group) of three or more independent studies were presented (*p < 0.05 versus control group). Full-length blots are presented in Supplementary Figure 7c–f. (**D**) Co-immunoprecipitation revealed physical interactions between XBP1u, s and FoxO1 in auditory cells. Data presented are representative of three independent experiments.

In order to confirm the interaction between XBP1 and FoxO1 in auditory cells, we examined the expression of FoxO1 in tunicamycin-treated XBP1 KD cells and XBP1u, s in tunicamycin-treated FoxO1 KD cells. Interestingly, according to Fig. 7A, in control siRNA group, the FoxO1 expression level was getting lower when the treatment time of tunicamycin was getting longer. In XBP1 siRNA group, the FoxO1 expression level stayed similar regardless of treatment time of tunicamycin stating that without XBP1, tunicamycin-induced ER stress failed to suppress FoxO1 expression. On the other hand, the expression of XBP1u peaked at 24 h, and that of XBP1s peaked at 12 h in tunicamycin-treated FoxO1 KD cells (Fig. 7B and C). There were no significant differences observed between si-control and FoxO1 KD cells.

Next, we conducted co-immunoprecipitation to detect direct interaction between XBP1 and FoxO1. Co-immunoprecipitation revealed physical interactions between XBP1u, s and FoxO1 in auditory cells (Fig. 7D). These results suggest that XBP1 regulates the FoxO1 expression, whereas FoxO1 did not affect the induction of XBP1u and XBP1s in auditory cells under ER stress condition.

## Discussion

In this study, we revealed that XBP1 mRNA splicing is implicated in the induction of autophagy in auditory cells through the transcriptional regulation of FoxO1 under ER stress condition. Knocked down of XBP1, IRE1α or FoxO1 by siRNA in auditory cells significantly blocked the expression of LC3-II (Figs 4D, 5B and 6D). XBP1 deficiency in auditory cells regulates FoxO1 level (Fig. 7A and D). These results suggest that the interaction of XBP1 and FoxO1 regulated the ER stress-induced autophagy in auditory cells.

First, we confirmed the expression of protein XBP1 that showed different dynamic states as compared with the level of XBP1 mRNA (Fig. 2B,C,F and G). A previous study reported that protein XBP1u interacts with XBP1s as a negative feedback regulator by proteasome-mediated degradation during the later phase of ER stress in the HeLa cells^56^. Our results also suggest that protein XBP1u in the cytoplasm interacts with XBP1s in the nucleus of the auditory cells under ER stress, functioning as a negative feedback regulator. In addition, we speculated the following points. Upon ER stress, IRE1α was up-regulated and activated by phosphorylation (Fig. 2A). The activated IRE1α spliced XBP1u mRNA into XBP1s mRNA, from which protein XBP1s was translated and then translocated into the nucleus during the early phase of ER stress (12 h after treatment) (Fig. 2E,F and G). Protein XBP1s binds to the UPR element and activates transcription of the gene required for the UPR and ERAD synthesis. In the later phase of ER stress (24 h after treatment), it was found that although XBP1 mRNA splicing continues, protein XBP1u increases and begins regulating protein XBP1s negatively to calm down UPR (Figs 2B,C and 7D). Taken together, our data indicates that ER stress induced IRE1α-mediated XBP1 mRNA splicing, and protein XBP1s play a major role as a transcriptional factor to alleviate ER stress by negative regulation of XBP1u in the auditory cells.

Autophagy is one of the major protein degradation systems for the maintenance of cellular homeostasis in response to various stresses such as nutrient deprivation^60^. A number of previous reports have already established the link between ER stress and the induction of autophagy^61–63^. A recent study demonstrated that XBP1 mRNA splicing is involved in the regulation of autophagy in endothelial cells^15^. In our study, the level of LC3-II, which is necessary for the process of autophagosome formation, was upregulated and peaked at 24 h after tunicamycin treatment (Fig. 3A and B)^64–66^. Furthermore, the largest number of autophagosome was observed in the cytoplasm at 24 h after tunicamycin treatment (Fig. 3C and D). These results indicate that autophagy was induced in auditory cells at 24 h after the treatment of tunicamycin.

As shown in Fig. 3E, the size of ER increased time-dependently in tunicamycin-treated cells. The mild expansion of ER containing materials like lipid was confirmed at 24 h after treatment, which indicates UPR induction.

Several reports have suggested that ER stress, including tunicamycin treatment, leads to ER expansion^67, 68^. ER expansion through UPR-mediated activation of lipid biosynthesis alleviates ER stress caused by providing additional ER surface area and luminal space while the continuous ER expansion results in cell death^69^. The presence of autophagosome and mild ER expansion at the same time also states the strong correlation between UPR and autophagy. At 48 h after treatment, the number of autophagosomes decreased, and on the other hand, ER expansion was much bigger (Fig. 3D and E). Interestingly, both the expression of XBP1s and LC3-II decreased at 48 h after treatment (Figs 2C and 3A). In other words, autophagy was also impaired at 48 h after treatment. This suggests that ER stress overtakes autophagy response and results in cell death, which means the breakdown of the cytoprotective system in the auditory cells. The marked decrease of cell viability also supports the breakdown of the cytoprotective system at 48 h after treatment (Fig. 1A and B).

Then we knocked down the expression of XBP1 or IRE1α by siRNA in order to investigate the relationship between the UPR and the induction of autophagy in auditory cells. As expected, the expression of LC3-II was significantly blocked in tunicamycin-treated XBP1 or IRE1α KD cells (Figs 4D and 5B). As shown in Figs 4E and 5C, tunicamycin-treated XBP1 or IRE1α KD cells showed a significant decrease in cell viability. Furthermore, TEM revealed that the number of autophagosomes was significantly decreased, and a number of aggregates existed in the cytoplasm of tunicamycin-treated XBP1 KD cells (Fig. 4F and G). These results suggest that XBP1 knockdown caused autophagy impairment in auditory cells. Taken together, our results paradoxically demonstrated that XBP1 mRNA splicing induced autophagy in auditory cells under ER stress condition, and ER stress-induced autophagy serves as a cell survival mechanism to protect against tunicamycin-induced cell death.

As previously mentioned, ER stress causes ER expansion in auditory cells. Previous studies also reported that XBP1s induces ER expansion by the augmentation of ER protein gene expression and lipid biosynthesis in fibroblasts^70, 71^. In the present study, the size of ER was significantly decreased in XBP1 KD cells as compared to si-control cells (Fig. 4H). Our data suggests that XBP1 induces ER expansion under ER stress condition in auditory cells. In addition to inducing UPR and autophagy in auditory cells, the mechanism of XBP1 also contributed to cell survival by augmenting ER capacity to alleviate ER stress.

It has been reported that FoxO1 is involved in the induction of autophagy through cytosolic or transcriptional activity in several cell lines^51–53^. In the present study, we found that FoxO1 was involved in the induction of autophagy because the expression of LC3-II was significantly blocked in tunicamycin-treated FoxO1 KD cells (Fig. 6D). A recent report showed the interaction between FoxO1 and XBP1. Zhao *et al*. reported that XBP1u directly interacted with FoxO1 and mediated the degradation of FoxO1 by 20S proteasome in the cytoplasm^54^. On the other hand, Zhou *et al*. reported that XBP1s negatively regulates FoxO1 by proteasome-mediated degradation in pancreatic β cells^55^. Based on these studies, we made a hypothesis that the interaction between XBP1 and FoxO1 existed in auditory cells. In order to investigate the relation of XBP1 and FoxO1, we analyzed the expression of FoxO1 in tunicamycin-treated XBP1 KD cells, and XBP1u, s in tunicamycin-treated FoxO1 KD cells. Tunicamycin-treated XBP1 KD cells showed the FoxO1 expression level stayed similar regardless of treatment time of tunicamycin, whereas tunicamycin-treated FoxO1 KD cells showed no significant differences in the XBP1u, s levels as compared to the si-control cells. These results suggest that tunicamycin-induced ER stress failed to suppress the FoxO1 expression without XBP1. However, FoxO1 did not affect the induction of XBP1u, s in auditory cells under ER stress condition. Furthermore, the direct interactions between XBP1u, s and FoxO1 were observed (Fig. 7D).

**Figure 8:**
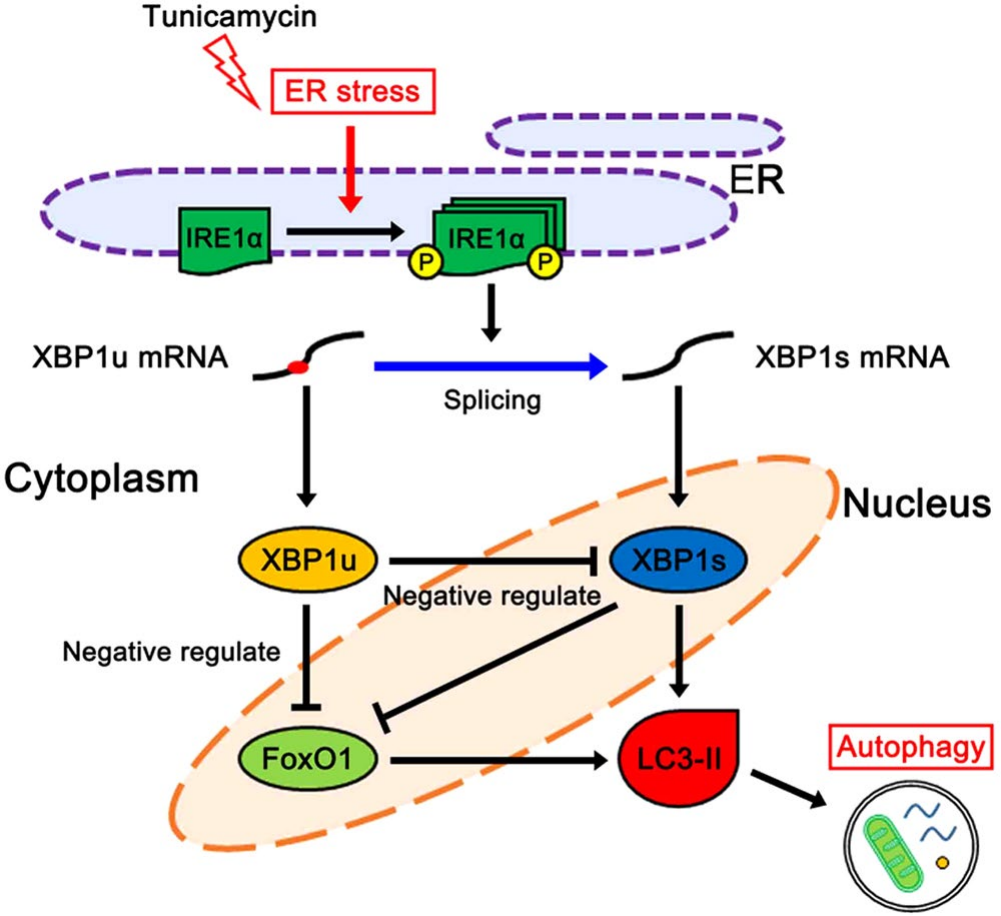
XBP1 regulated autophagy through the interaction of FoxO1 in auditory cells under ER stress condition. A schematic model shows XBP1 splicing induces ER stress-induced autophagy through the transcriptional regulation of FoxO1 in auditory cells.

In conclusion, these results indicated that XBP1 regulated autophagy through the interaction of FoxO1 in auditory cells under ER stress condition (Fig. 8). To our knowledge, this is the first-ever study to show the association between ER stress and autophagy regulated through the interaction of XBP1 and FoxO1 in the auditory cells. However, our findings explained how ER stress and autophagy affected the auditory cellular function. The study also contributed to pathology underlying hearing impairment. However, further studies need to be done for complete elucidation of the pathogenesis of hearing impairment.

## Materials and Methods

### Reagents and antibodies

Tunicamycin was purchased from Sigma-Aldrich (St. Louis, MO, USA). Chloroquine and bafilomycin A1 were from MBL (Nagoya, Japan). The following primary antibodies were purchased from Cell Signaling Technology (Danvers, MA, USA): anti-IRE1α, anti-XBP1s, anti-FoxO1, anti-full length and cleaved caspase-3, anti-HDAC1, anti-GAPDH and anti-β-actin antibodies. The anti-XBP1u antibody was obtained from Santa Cruz Biotechnology, Inc (Dallas, TX, USA). Anti-LC3 antibody was purchased from MBL. Anti-p-IRE1α was from Abcam (Cambridge, UK). Anti-goat mouse and anti-rabbit antibodies were from Santa Cruz Biotechnology, Inc and Cell Signaling Technology, respectively. Small interfering RNA (siRNA) for XBP1, IRE1α and control siRNA were from Santa Cruz Biotechnology, Inc. FoxO1 siRNA were from Cell Signaling Technology. Cytoplasmic & Nuclear Protein Extraction Kit was from 101 Bio (Palo Alto, CA, USA).

### Cell culture and culture condition

The HEI-OC1 cell line was kindly provided by F. Kalinec (UCLA, Los Angeles, CA, USA)^72^. The cells were maintained in a high-glucose Dulbecco’s modified Eagle’s medium (DMEM; Gibco, Grand Island, USA) supplemented with 10% fetal bovine serum (FBS; Invitrogen, Carlsbad, CA, USA), 1% penicillin-streptomycin (Gibco), and 50 U/ml mouse IFN-γ (Merck Millipore, Darmstadt, Germany). HEI-OC1 cells were cultured under the following permissive conditions: 33 °C and 10% CO_2_.

### Cell viability assay

HEI-OC1 cells (5 × 10^4^ cells/ml/well of 24 well plates) were incubated with various concentrations of tunicamycin (5, 50 and 100 µg/ml) at 33 °C for 12, 24 and 48 h, and then suspended in an equal volume of 0.4% trypan blue (Gibco). Dead (blue) and live (clear) cells were counted with a hemocytometer. The percentage of viability was defined as percent live per total cells.

### Detection of cell death by fluorescence microscopy and flow cytometry

Cells death was measured by Annexin V-FITC Apoptosis Detection Kit (Abcam) according to the manufacturer’s instructions. Briefly, the cells were seeded in 24 well plates at the density of 5 × 10^4^ cells/ml for fluorescence microscopy and 6 × 10^5^ cells/ml in 60 mm culture dishes for flow cytometry, and incubated with 50 µg/ml of tunicamycin at under the permissive conditions for 0, 12, 24 and 48 h. After removing culture medium, cells were washed with binding buffer. Then, 5 µl of Annexin V-FITC and 5 µl of propidium iodide (PI) were added and incubated at room temperature for 5 min in the dark. For imaging, the cells were observed using a fluorescence microscope (BZ-X710, KEYENCE, Osaka, Japan). For flow cytometry, the cells were acquired by flow cytometer (Gallios Flow Cytometer, Beckman Coulter, Brea, CA, USA) and analyzed using FlowJo software (FlowJo, LLC, Ashland, OR, USA). Cells that were PI negative and Annexin V negative were considered healthy, cells that were PI negative and Annexin V positive were considered early apoptotic, and cells that were positive to both PI and Annexin V were considered late apoptotic and necrotic.

### Protein extraction and Western blot analysis

Western blotting was performed as follows. The samples (10 µg) were subjected to electrophoresis on sodium dodecyl sulfate-polyacrylamide gels (4–12%, 12%) for 90–120 min at 20 mA and then transferred onto PVDF membranes using iBlot (Life Technologies, Carlsbad, CA, USA). The membrane was incubated overnight at 4 °C in the presence of primary antibodies at dilutions of 1:1000-1:3000 in TBS-T. After three washes with TBS-T, the membrane was incubated with the corresponding species-appropriate secondary antibodies at a dilution of 1:2000-1:3000 in TBS-T for 1 h. Then the immunoreactive bands on the membrane were visualized using LAS-4000 mini (FUJIFILM, Tokyo, Japan).

### Co-immunoprecipitation

Co-immunoprecipitation was performed Dynabeads^®^ Protein G Immunoprecipitation Kit (Thermo Fisher Scientific, Waltham, MA, USA) according to the manufacturer’s instructions. Cells were incubated with 50 µg/ml tunicamycin for 24 h and then lysed in RIPA buffer (Wako, Osaka, Japan) with protease inhibitor (Nacalai, Tokyo, Japan). Lysates were incubated overnight on carousal 4 °C with first antibody. After addition of beads, the incubation continued for 90 min. Immunoprecipitates were extensively washed 3 times using washing buffer and eluted with SDS sample buffer by boiling 10 min at 70 °C. Subsequently, samples were subjected to Western blot analysis.

### Transmission electron microscopy

The cells were fixed with 2.5% glutaraldehyde in 0.1 M cacodylic buffer solution (pH 7.4) overnight, post-fixed with 1% osmium tetroxide in 0.1 M cacodylic buffer solution (pH 7.4) for 2 h, dehydrated through graded ethanol, and embedded in Quetol-812. Ultrathin sections were cut with a diamond knife on an ultramicrotome (ULTRACUT UCT, Leica, Wien, Austria). The thin sections were stained with uranyl acetate and lead citrate and observed using a transmission electron microscope (JEM-1200EX, JEOL, Tokyo, Japan).

### Transient siRNA transfection

Small interfering RNA (siRNA) against XBP1, IRE1α (Santa Cruz Biotechnology, Inc), FoxO1 (Cell Signaling Technology) and control siRNA (Santa Cruz Biotechnology, Inc) were used for knockdown of the XBP1, IRE1α, and FoxO1 genes. The HEI-OC1 cells were cultured to achieve 60–80% confluency. Then the cells were transfected with siRNA according to the manufacturer’s protocol. After 48 h of transfection, the cells were treated as designed.

### RT-PCR

Total RNA was extracted with RNeasy Mini kit (QIAGEN, Hilden, Germany). cDNA was synthesized from RNA with Oligo (dT) 12–18 Primer (Invitrogen) using SuperScript III Reverse Transcriptase (Invitrogen) and RNase Inhibitor (Ambion, Austin, TX, USA). The PCR products were electrophoresed using a microchip electrophoresis system (MCE-292 MultiNA, Shimadzu, Kyoto, Japan) and quantified by MultiNA Viewer software. PCR products were introduced into each microchip (88 × 50 μm) and electrophoresis was performed with capillary electrophoresis buffer. The migrating nucleic acids were detected by fluorescence detection with SYBR Gold. The size standard curve to detect the size of the PCR product was generated from the migration time and the fragment size of ladder. The DNA concentration in each peak was quantified from the sample peak area and upper marker (UM) peak area. Measurement results were expressed by converting into classical electrophoresis gel-like image^73, 74^. The primers for XBP1 and GAPDH were as follows; for XBP1 5′-GAACCAGGAGTTAAGAACACG-3′ and 5′-AGGCAACAGTGTCAGAGTCC-3′; for GAPDH, 5′-CCATCACCATCTTCCAGGAG-3′ and 5′-ACAGTCTTCTGGGTGGCAGT-3′. The PCR conditions were: 94 °C for 2 min; 35 cycles of 98 °C for 15 sec, 55 °C for 10 sec, 68 °C for 30 sec and 68 °C for 3 min.

### Statistical analysis

All the data were expressed as mean ± S.D. Statistical analysis was carried out by one-way variance and Student’s *t*-test. A P-value less than 0.05 indicated statistical significance.

## Electronic supplementary material

Supplementary Information
