# Supplementary material for: XBP1-FoxO1 interaction regulates ER stress-induced autophagy in auditory cells
Source: Sci Rep. 2017 Jun 30;7:4442. doi: 10.1038/s41598-017-02960-1 (PMC5493624; doi:10.1038/s41598-017-02960-1)
Supplement: Supplementary file 1 — Supplementary Information [file 41598_2017_2960_MOESM1_ESM.pdf]

**Supplementary Information**

# XBP1-FoxO1 interaction regulates ER stress-induced autophagy in auditory cells

Akihiro Kishino[1], Ken Hayashi[2], Chiaki Hidai[3], Takeshi Masuda[1], Yasuyuki Nomura[1] and Takeshi Oshima[1*]

[1] Department of Otolaryngology, School of Medicine, Nihon University, Tokyo 173-8610, Japan
[2] Department of Otolaryngology, Kamio Memorial Hospital, Tokyo 101-0063, Japan
[3] Department of Physiology, School of Medicine, Nihon University, Tokyo 173-8610, Japan

*Corresponding author: Dr. Takeshi Oshima, Department of Otolaryngology, School of Medicine, Nihon University, Postal address: 30-1, Oyaguchi Kami-cho, Itabashi-ku, Tokyo, Japan 173-8610, Phone: +81-3-3972-8111, Fax: +81-3-3972-1321, E-mail: takeosh55@gmail.com

## Supplementary Figure 1.

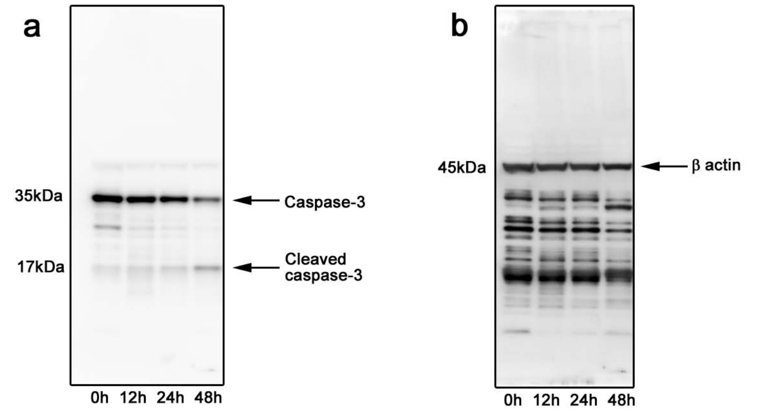


## Supplementary Figure 2.

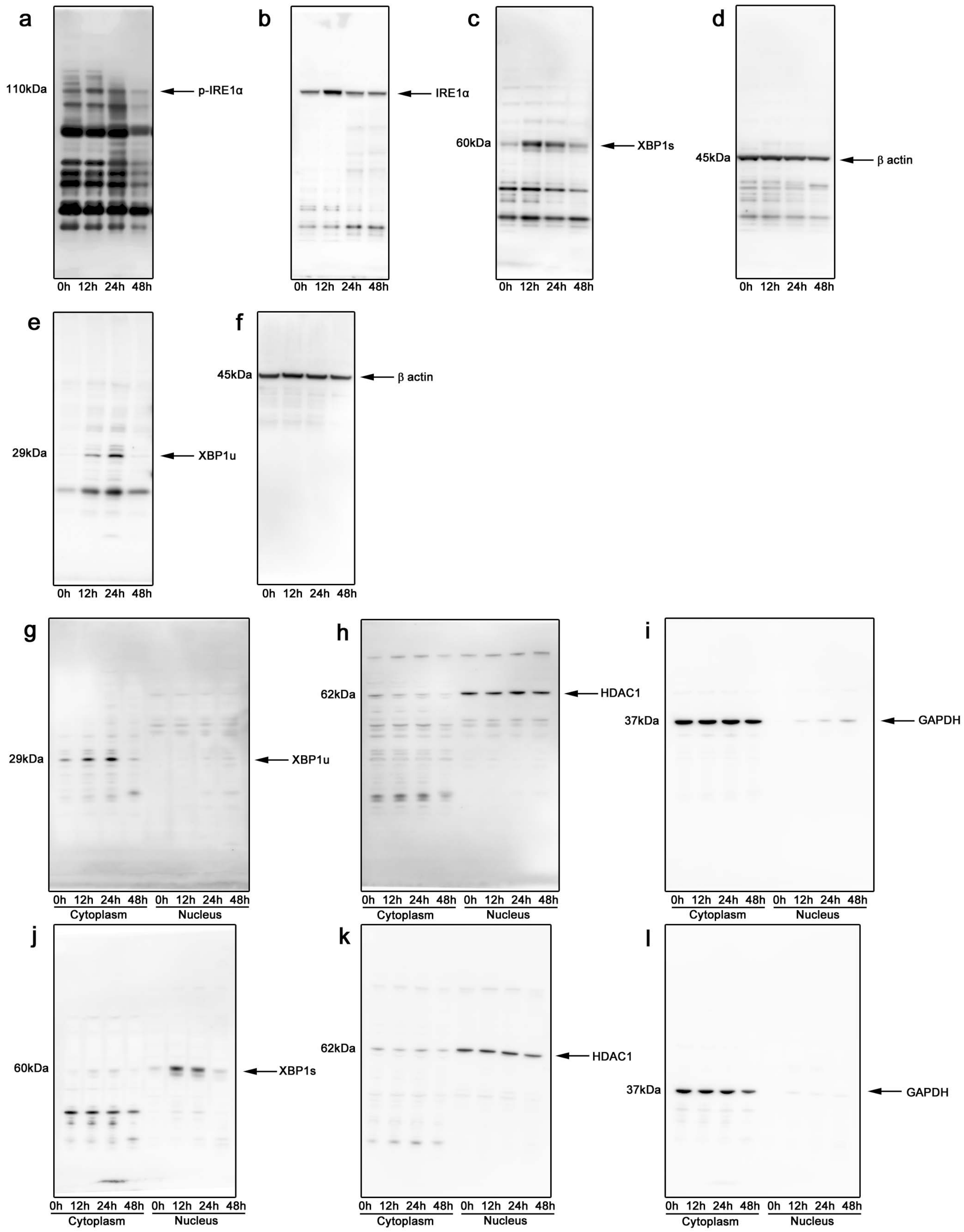

## Supplementary Figure 3.

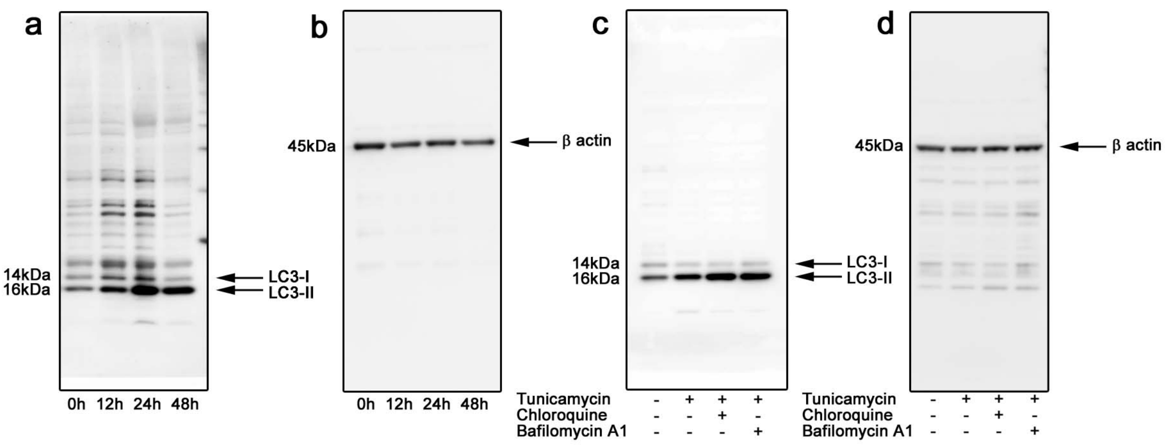


## Supplementary Figure 4.

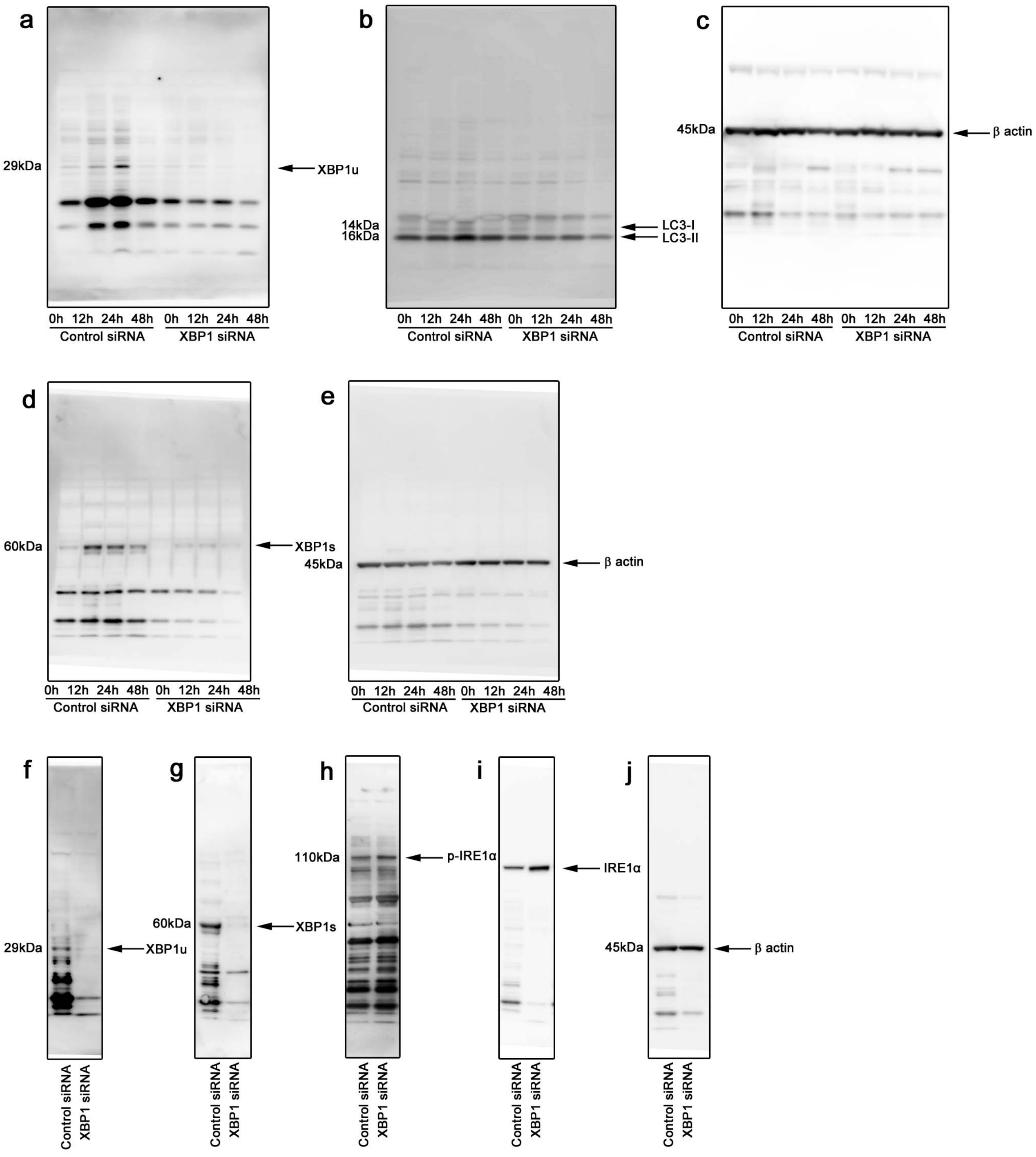

## Supplementary Figure 5.

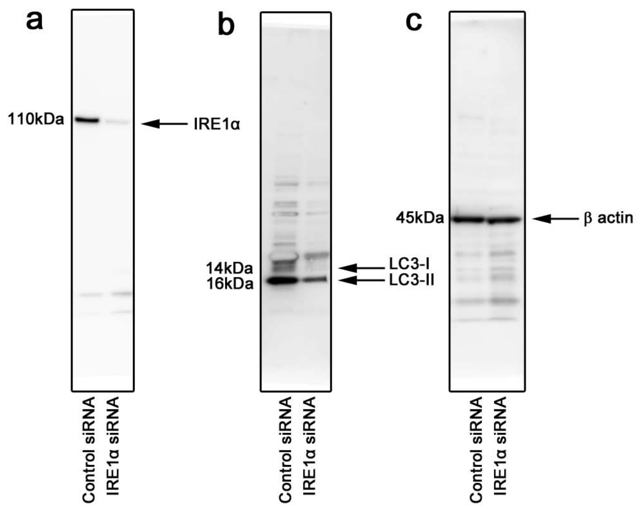


## Supplementary Figure. 6

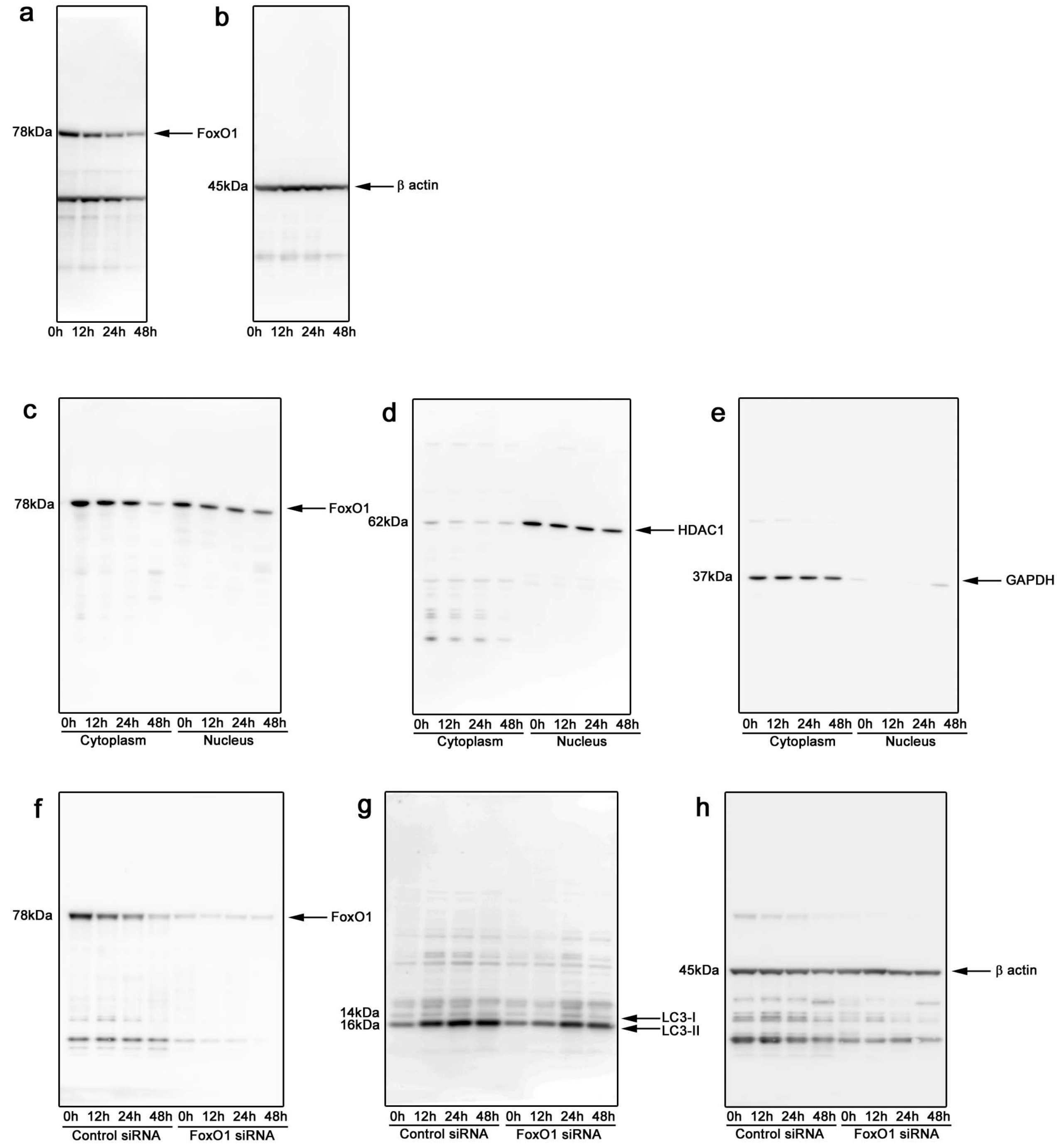

## Supplementary Figure. 7

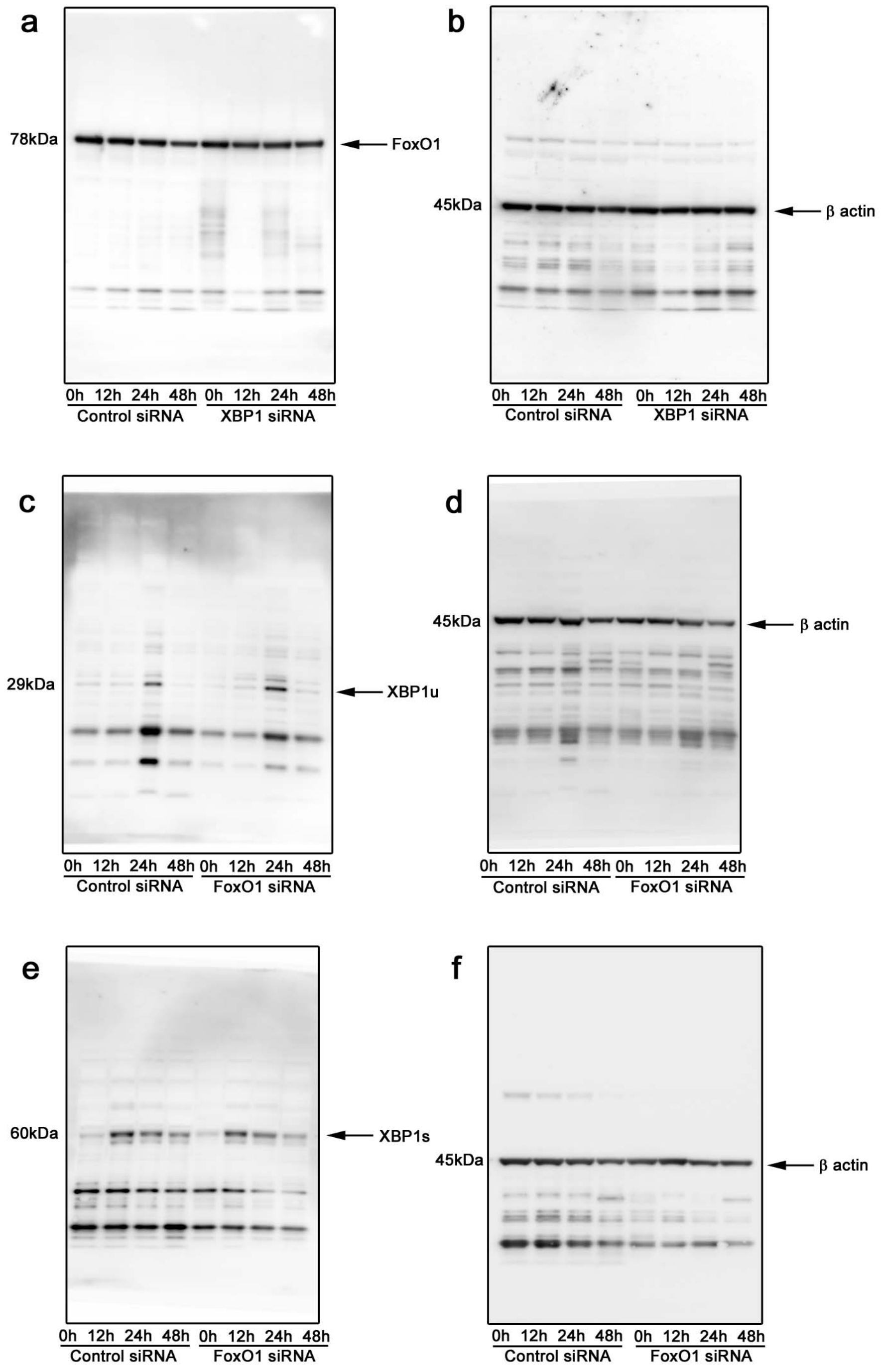

**Supplementary Figures S1-7.**

Uncropped version of blots displayed in main figure 1F (Fig. S1 a and b), 2A (Fig. S2 a, b and d), 2B (Fig. S2 e and f), 2C (Fig. S2 c and d), 2D (Fig. S2 g, h and i), 2E (Fig. S2 j, k and l), 3A (Fig. S3 a and b), 3B (Fig. S3 c and d), 4A (Fig. S4 a and c), 4B (Fig. S4 d and e), 4C (Fig. S4 f, g, h, i and j), 4D (Fig. S4 b and c), 5B (Fig. S5 a, b and c), 6A (Fig. S6 a and b), 6B (Fig. S6 c, d and e), 6C (Fig. S6 f and h), 6D (Fig. S6 g and h), 7A (Fig. S7 a and b), 7B (Fig. S7 c and d) and 7C (Fig. S7 e and f).
